# Supplementary material for: Interfacial Behavior of Solid- and Liquid-like Polyelectrolyte Complexes as a Function of Charge Stoichiometry
Source: Polymers (Basel). 2021 Nov 7;13(21):3848. doi: 10.3390/polym13213848 (PMC8588307; doi:10.3390/polym13213848)
Supplement: Supplementary file 1 [file polymers-13-03848-s001.zip › polymers-1434331-supplementary.pdf]

Interfacial Behavior of Solid- and Liquid-like Polyelectrolyte Complexes as a Function of the Charge Stoichiometry

Hongwei Li,[1,2] Martin Fauquignon[1,2] Marie Haddou, [1,2], Christophe Schatz,[2] Jean-Paul Chapel[1]

[1]*Centre de Recherche Paul Pascal (CRPP), UMR CNRS 5031, Univ. Bordeaux, 33600 Pessac, France.*

[2]*Univ. Bordeaux, CNRS, Bordeaux INP, LCPO, UMR 5629, F-33600 Pessac, France*

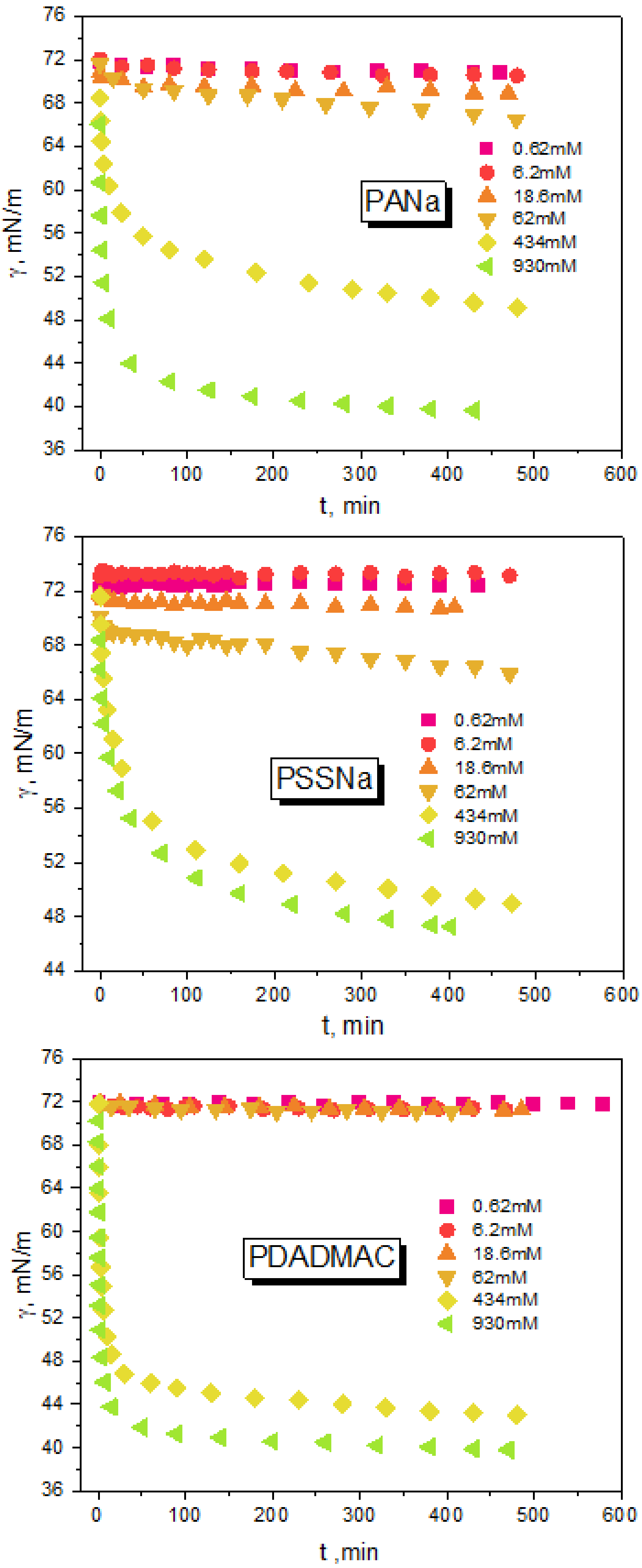

Figure S1. Dynamic surface tension (γ) of PANa, PSSNa and PDADMAC solutions at different concentrations (in repetitive units) determined by pendant-drop measurements

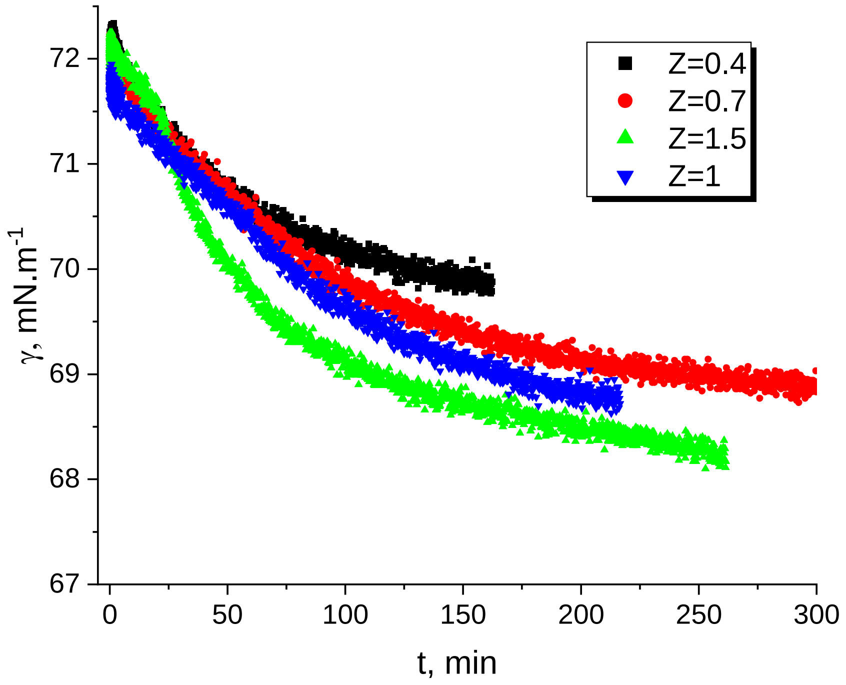

Figure S2. Dynamic surface tension (γ) of PDADMAC/PANa complexes prepared *off* the stoichiometry at Z=0.4, 0.7, 1.0 and 1.5 from 1 mM PE stock solutions.
